# Normative longitudinal EEG recordings during sleep stage II in the first year of age

**DOI:** 10.1038/s41597-024-03606-4

**Published:** 2024-07-17

**Authors:** Thalía Harmony, Gloria Otero-Ojeda, Eduardo Aubert-Vázquez, Thalía Fernández, Lourdes Cubero-Rego

**Affiliations:** 1 Neurodevelopment Research Unit at the Instituto de Neurobiología, Universidad Nacional Autónoma de México; Juriquilla, Querétaro, CP.76230 México; 2 Facultad de Medicina de la Universidad del Estado de México, Toluca, México; 3 Cuban Neuroscience Center, Havana, Cuba; 4 Laboratorio de Psicofisiología, Instituto de Neurobiología, Universidad Nacional Autónoma de México; Juriquilla, Querétaro, CP.76230 Mexico

**Keywords:** Neonatal brain damage, Paediatric research

## Abstract

The electroencephalogram (EEG) is a fundamental diagnostic procedure that explores brain function. This manuscript describes the characteristics of a sample of healthy at-term infants. One hundred and three (103) infants from Mexico between 15 days and 12.5 months of age were recorded during physiological sleep. Referential EEG recordings were obtained using linked ear lobes as reference. The amplifier gain was 10,000, the bandwidth was set between 0.3 and 30 Hz, and the sample rate was 200 Hz. Sample windows of 2.56 s were marked for later quantitative analysis. To our knowledge, this is the first dataset of normal infants during the first year of age.

## Background & Summary

The electroencephalogram (EEG) is a fundamental diagnostic instrument. Visual inspection of the EEG trace has been used for diagnosis since the beginning of the last century, and today, it is a routine examination like an electrocardiogram or a blood count. It is economical and noninvasive and can be used for all ages, from neonates to old patients. A promising development in EEG research is using artificial intelligence (AI) as an advanced signal-processing tool to look for biomarkers that may identify sex, age, and diverse pathologies. For this reason, the appearance of public data sets has become very useful. Large, standardized raw EEG across the lifespan databases are available^1–3^.

However, although these databases cover a broad band of ages (from children to senescence), they do not include infants. This manuscript accompanies the data set of a longitudinal EEG dataset^4^ of 103 term healthy infants during the first year of age.

## Methods

The Ethics Committee of the Instituto de Neurobiología of the Universidad Nacional Autónoma de México and the Medicine Faculty of the Universidad Autónoma del Estado de México approved this study, which also complies with the Ethical Principles for Medical Research Involving Human Subjects established by the Declaration of Helsinki. Informed written parental consent for participation in this study was obtained for all subjects.

The reference dataset^4^ is considered part of the subjects reported by^5–7^.

**Fig. 1:**
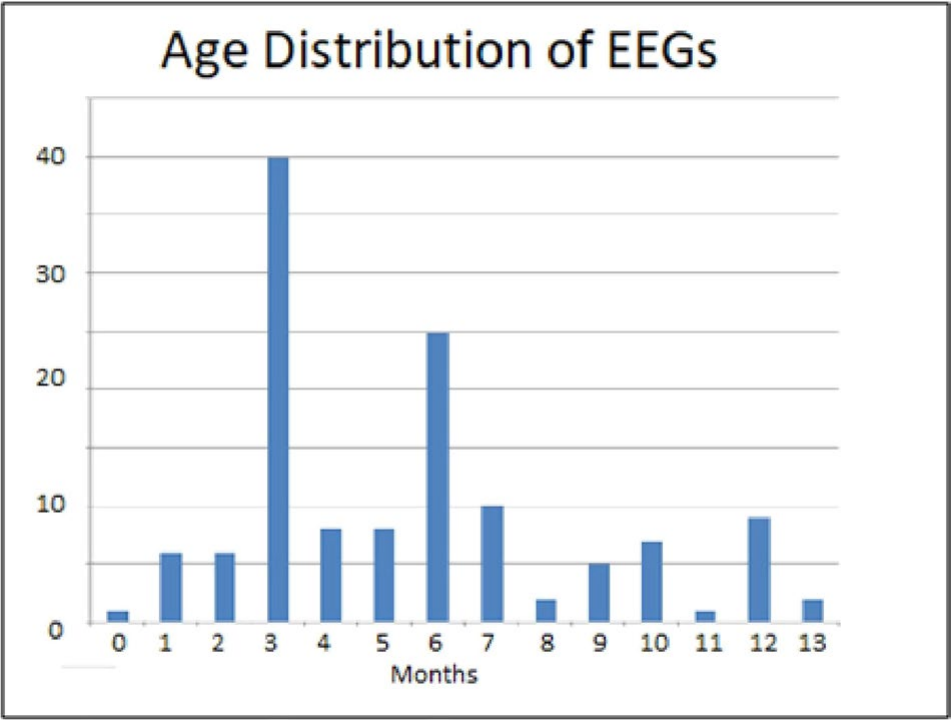
Age distribution of the sample.

In these papers, we described how the recordings were done. However, to help readers,we again include this information in this manuscript.One hundred and three (103) infants between 15 days and 12.5 months of age from two states in the central region of Mexico (Querétaro and Estado de México) were studied. Figure 1 shows the age distribution of the sample.

To include each case in the study, we followed the criteria described by Otero^5^: 1. Normal delivery at birth; 2. Birth weight between 2500 g and 3900 g; 3. Apgar ≥8 at the first minute and ≥9 at 5 min after birth; 4. No background of pre- or perinatal risk of CNS damage; 5. Physical examination within normal limits; 6. Normal neurological and pediatric examination results. 7. Normal psychomotor development as evaluated by the Bayley II Test^8^.

Recording the EEG of infants during wakefulness may be very difficult because of the presence of movement artifacts. Usually, the acquisition is obtained during physiological sleep. Several books describe the EEG during the first year of age in healthy term infants during sleep^9–11^.

The slow sleep stage II is characterized by delta waves during the first year of life, with intermixed theta, alpha, and beta activity of smaller amplitude than the delta waves. During sleep stage II, a decrease in delta waves and an increase in theta waves are observed during the first year^12^. In this stage of sleep, from 2-3 months, sleep spindles appear; these have a frequency of 12 to 15 Hz, at 14 Hz more frequently. The spindles of infants have a negative sharp component and are maximal at the central and parietal regions. The spindle trains are of short duration and low voltage initially, and with age, the amplitude and duration of the spindle trains increase^13–15^.

Another characteristic of sleep stage II is the observation of Vertex waves and K complexes, usually detected around 5-6 months of age and with frontocentral localization. Vertex waves are slow transients focal at Cz and can also be observed at C3 and C4. K complexes are sharp, slow waves frequently associated with spindle bursts. They are midline and predominantly central in origin and typically bilaterally symmetrical. They are often seen as a response to an abrupt auditory stimulus, but they also appear spontaneously, without external stimulation^9–12^.

The study was performed in a dimly lit, soundproof room while infants slept. We used a polyester cap with surface electrodes distributed according to the 10–20 International System (Fp1, Fp2, F3, F4, C3, C4, P3, P4, O1, O2, F7, F8, T3, T4, T5, T6, Fz, Cz, Pz). Referential EEG recordings were taken during spontaneous quiet sleep, stage II, using linked ear lobes as reference. We take care that impedances were at or below 5000 Ω. During recordings, infants were on their mother´s lap.

We tried to obtain 30 minutes of recordings. However, this duration was not possible in many cases because infants woke up, and the presence of artifacts made it impossible to continue the recordings. Although the duration of the recordings was variable, they had a median duration of approximately 8 minutes.

A digital electroencephalograph (Medicid 3E) was used with a gain of 10,000, the amplifier bandwidth was set between 0.3 and 30 Hz, and the sample rate was 200 Hz. As Mexico’s main line power frequency is 60 Hz, we use a filter around 60 Hz.

Two experienced clinical neurophysiologists (TH and GO-O) observed all recordings to confirm their usefulness and the sleep stage, and windows of 2.56 s were marked for further quantitative analyses.

## Data Records

As previously indicated, the reference dataset^4^ is considered part of the subjects reported by^5–7^.The dataset obtained was formatted as a BIDS dataset. BIDS (Brain Imaging Data Structure) is the new standard for organizing and describing neuroimaging datasets (MRI, MEG, EEG, iEEG, NIRS, PET) and behavioral information^3,16^. To fulfill the requirements for BIDS, we developed a methodology with the following steps:

### Anonymization of EEG recordings

The application anomplg.exe (Windows 32 bits) was used to erase all the personal information stored in the EEG recordings in Neuronic (manufacturer of the Medicid 3-E digital electroencephalograph systems used to acquire the EEGs) format, which could facilitate the identification of the participants. This application generated a secured copy of the personal information before its elimination. In this step, the following information is removed from the subject’s data: the subject’s name, the subject’s birthdate, the subject’s record date, the subject’s referring physician, and the site where the EEG is recorded. The personal information deleted makes the participant’s identification impossible. Only age and gender at EEG recording time are kept. Age is a very relevant variable for EEG interpretation and processing.

### Conversion of EEG recordings to BIDS

The application plg2bids.exe (Windows 32 bits) was used to read the original EEG recordings in Neuronic format and convert them into BIDS. This application is designed to read either individual EEG recordings or folders with multiple recordings and can update a current BIDS structure with new recordings.

Due to the normative nature of the dataset described, it was decided to include annotations filled by expert clinical neurophysiologists on the EEG raw data as part of the dataset annotations. These annotations could be used to carry out, for instance, quantitative analysis of the EEG. The annotations consisted of selecting EEG windows/epochs/segments with sufficient quality for subsequent processing. Therefore, these annotations, the product of processing after data collection, must be stored in the ‘\derivatives’ folder of the dataset. For each subject of the dataset is saved to the file sub-XXXXXX_ses-X_task-EEG_annotations.tsvt he following fields for each marked window:

onset: start of analysis window in seconds.

duration: duration of the analysis window in seconds.

label: window type identifier

### Validation of the BIDS structure

The final step was validating the BIDS structure using the web BIDS validator. https://bids-standard.github.io/bids-validator/.

This is the first longitudinal EEG dataset^4^ of infants during physiological sleep during the first year of age.

**Fig. 2:**
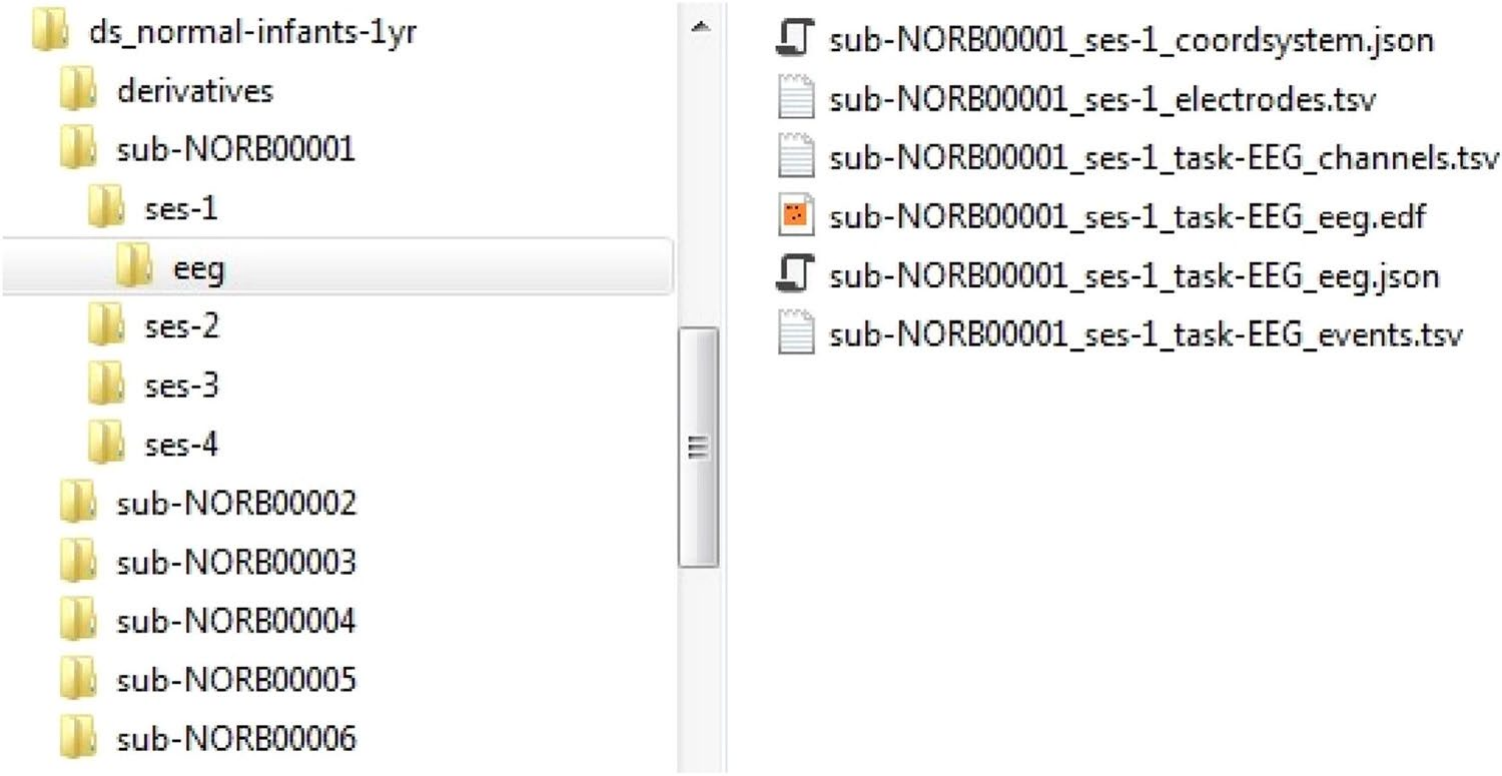
Dataset folder structure.

The dataset^4^ is available at the OpenNeuro repository (10.18112/openneuro.ds004577.v1.0.1) and, as stated in the previous section, is formatted as a BIDS dataset. Figure 2 shows a screenshot of the BIDS dataset characteristic file and folder structure using the dataset presented in this paper as an example. The left panel of the figure shows the folders for each subject, including the different sessions. The right panel shows the contents of the \eeg folder where the files related to the EEG modality are stored. All the files and folder names were selected, following the BIDS specification guidelines (https://bids-specification.readthedocs.io/en/stable/). Below is a brief description of the files.

*_coordsystem. json: used to specify the fiducials, the location of anatomical landmarks, and the coordinate system and units in which the position of electrodes and landmarks is expressed.

*_electrodes.tsv: gives the location of EEG electrodes.

*_channels.tsv: gives information about the channels used.

*_eeg.edf: contains the raw EEG data in EDF format.

*_eeg. json: contains metadata related to the EEG recording.

*_events.tsv: describe the timing and other properties of events recorded during the EEG acquisition.

## Technical Validation

**Fig. 3:**
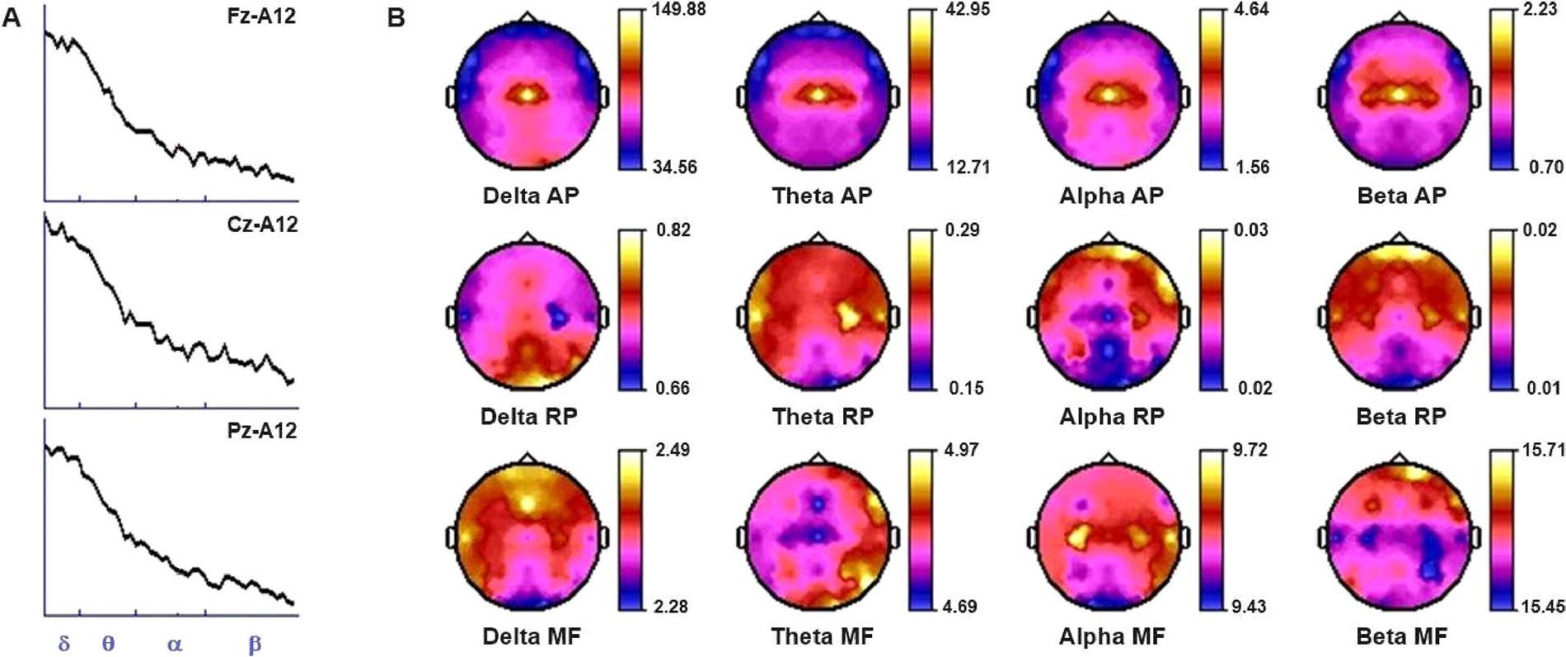
Example of some spectral values of a subject. A: Power spectra at the midline leads (Fz, Cz, and Pz); the frequency bands (delta, theta, alpha, and beta) are indicated below. B: Maps of three spectral measurements are shown in each row (AP: absolute power, in mV2/Hz; RP: relative power; and MF: mean frequency, in Hz); the columns represent the delta, theta, alpha, and beta frequency bands.

For the quality control of the EEG, all recordings were observed by two experienced clinical neurophysiologists (TH and GAO) to confirm their usefulness and windows of 2.56 s were marked for further quantitative analyses. Figure 3 shows some results from quantitative analysis for a subject in the dataset.

The BIDS dataset described in this paper was validated using the web BIDS validator. https://bids-standard.github.io/bids-validator/.

## Usage Notes

Each participant was assigned one ID with the structure NORBxxxxx 4 characters identifying “NORmal Babies” and five digits indicating the number or order of the participant in the dataset. In the \derivatives folder of the BIDS dataset, information relevant to quantitative analysis is stored as annotations.

## Data Availability

The software used is at https://github.com/eduardo-aubert/BIDS-Conversion-Code. The codes are: 1) Anomplg.exe: to anonymize the EEG records, all the personal information stored in the EEG recordings, which could facilitate the identification of the participants, was deleted. 2) Plg2bids.exe: to read the original EEG recordings in NEURONIC format and convert them to BIDS structure.
